# The regional variation of a housing boom. Disparities of land prices in Austria, 2000–2018

**DOI:** 10.1007/s10037-022-00176-z

**Published:** 2023-01-23

**Authors:** Christian Reiner, Robert Musil

**Affiliations:** 1 Lauder Business School, Hofzeile 18–20, 1190 Vienna, Austria; 2 Institute for Urban and Regional Research, Austrian Academy of Sciences, Bäckerstraße 13, 1010 Vienna, Austria

**Keywords:** Land prices, Housing boom, Regional disparities, Spatial diffusion, Austria, Baulandpreise, Immobilienboom, Regionale Disparitäten, Räumliche Diffusion, Österreich

## Abstract

Debates accompanying the global housing boom have primarily focussed on the economic and social implications for urban housing markets. Against this background, this paper analyses the repercussions for regional land prices of a national housing boom in and beyond agglomerations. Convergence and divergence dynamics, regional price drivers, and spatial diffusion are investigated by examining average building-land prices of 95 Austrian regions between 2000 and 2018. The results indicate a clear increase in regional disparities in land prices, with the main rise taking place during a high price-growth period. Regions with high land prices are the main drivers of divergence, while a substantial number of peripheral regions with converging land prices were hardly affected by the national price boom. Land-price growth rates are positively affected by the number of households but negatively impacted by income growth, which points to a problematic decoupling of household income and land prices. Finally, the diffusion of the land-price boom occurs along the urban hierarchy as well as via neighbouring regions, confirming the ripple-effect hypothesis.

## Abstract (de)

Die wissenschaftliche Debatte über den globalen Immobilienboom fokussiert auf die ökonomischen und sozialen Implikationen von städtischen Wohnungsmarktdynamiken. Vor diesem Hintergrund untersucht dieser Beitrag die Auswirkungen eines Immobilienbooms auf die Baulandpreise sowohl in den urbanen Räumen als auch in den Regionen abseits davon. Dazu werden anhand der durchschnittlichen Baulandpreise in 95 österreichischen Regionen (politische Bezirke) zuerst Konvergenz- und Divergenzmuster analysiert. Weiters erfolgt eine Differenzierung der Preisdynamiken nach Indikatoren der regionalen Wirtschafts- und Sozialstruktur, sowie eine Analyse der regionalen Diffusionsmuster des Immobilienbooms. Die Ergebnisse weisen auf eine Zunahme der regionalen Disparitäten der Baulandpreise, die vor allem in der Periode des starken Preisanstiegs erfolgte, hin. Regionen, die schon am Beginn des Untersuchungszeitraumes von einem hohen Niveau der Baulandpreise gekennzeichnet waren, waren die zentralen Treiber der Divergenz. Umgekehrt existiert eine beträchtliche Zahl an peripheren Regionen, die vom nationalem Immobilien-Boom kaum betroffen waren, mit einem Trend zur Preis-Konvergenz. Die Wachstumsrate der Baulandpreise wird signifikant positiv durch die Haushaltszahl beeinflusst, umgekehrt zeigt sich für das Haushaltseinkommen ein negativer Effekt. Dieser Aspekt weist auf eine problematische Tendenz zur Entkoppelung von Einkommen und Baulandpreisen in vielen Regionen, auch abseits der Städte hin. Die räumliche Diffusion des Immobilienbooms erfolgt sowohl entlang der nationalen Städtehierarchie, als auch – entsprechend dem Ripple-Effekt – über benachbarte Regionen.

## Introduction

The global housing boom has provoked public debates which have predominantly focussed on urban agglomerations and the “housing question” regarding the affordability and inclusiveness of urban housing markets (e.g. Martin 2011; Holm 2009; Vollmer 2018; Wachsmuth and Weisler 2018; Aigner 2018). Little attention has yet been paid to temporal and spatial price dynamics beyond large urban agglomerations (rare exceptions include Blanco et al. 2016; Casolaro and Fabrizi 2018; Mariš 2021). Thus, the aim of this paper is to gain deeper insights into the regionally variegated effects of building land price developments in the context of a national housing price boom as experienced by the Austrian economy in the aftermath of the global financial crisis. According to data from the Austrian Central Bank, Austrian residential property prices stagnated in the years after 2000 but started to rise during the Great Recession, doubling between 2007 and 2020.

To shed light on the regional variation of this national housing boom, the empirical analysis in this paper is based on regional building-land prices as opposed to house prices. Regional average prices for land plots for detached houses (sized 600–800 m^2^) are used as an indicator for building-land prices. Analysing land prices can be motivated by several factors: Firstly, despite the long tradition of theoretical analysis of land prices in economic geography, there is a dearth of empirical research using data on land prices. Secondly, land is a more homogeneous good than houses or apartments are. Thirdly, compared to the building stock, land and land prices remain largely neglected in recent convergence debates (Özdilek 2011). Fourthly, land prices vary more over space than do prices for houses or apartments. For instance, the ratio between the most and the least expensive Austrian regions is about 30 for average land prices but only about 3 for average apartment prices. Thus, we argue that building-land prices are a more space-sensitive indicator for capturing regional disparities and price cycles than are price dynamics for single-family houses or apartments.

Investigating Austrian building-land prices is interesting due to Austria’s small-structured urban system (Lichtenberger 2002; ÖROK 2009), the decentral economy (Palme 1995) and the federally organised state (Kanonier and Schindelegger 2018). These factors are expected to result in a pronounced regional differentiation in land prices. In 2018, Austrian average building-land prices at the district level ranged between 1015 €/m^2^ and 34 €/m^2^. Urban agglomerations and tourism-intensive regions are the most expensive regions and the lowest land prices can be found in the north- and south-eastern economic periphery. With the exception of Vienna, building-land prices follow a pronounced west-east-price gradient. Further, the ownership structure of the Austrian housing market is characterised by a substantial degree of heterogeneity: high shares of private rental and of public housing dominate in urban markets, whereas owner-occupiers are more prevalent among suburban and rural markets. Finally, there are concerns about the dangers of a price bubble in Vienna (OeNB 2020), but there has been hardly any empirical research on (excessive) price dynamics beyond the Austrian capital (Schneider and Wagner 2015; Mundt and Wagner 2017; Van-Hametner and Zeller 2018).

This paper addresses the following three research questions, in accordance with the research gaps as outlined below: (1) Do regional building-land prices in Austria converge or diverge over time? (2) Which fundamental factors explain differences in regional building-land price growth rates? (3) Does the spatial diffusion of the building-land price boom occur alongside the urban hierarchy or towards neighbouring regions?

The paper is structured as follows: section two reviews the relevant literature and characteristics of Austrian land and housing markets. Section three presents data and methods. Section four, five and six comprises the empirical analysis and the final section discusses the empirical results as well as implications for future research.

## Determinants and disparities of land prices

Land is a basic good, a necessity for households and firms. According to Polanyi (2001), it is a fictitious commodity such as labour, i.e. it is not produced for sale on markets. Today’s capitalist land markets developed through the long and contested historical process of commodification. Hinting at the social relevance of land, Polanyi (2001) warns against social divisions if the availability of land is left to the whims of socially disembedded markets. Due to this and a number of other factors, land markets in developed countries are—albeit to a different degree—subject to rather comprehensive regulations (Cheshire 2013).

Land prices are influenced by spatial socio-economic developments and land prices in turn affect locational choices of households and firms, thereby shaping regional patterns of economic activity. Yet, real-estate market data predominantly rely on physical, built structures. Consequently, building land is seen as merged with the building stock, in which the land parcel is “hidden” (Özdilek 2011). This conceptual and empirical “invisibility” stands in contrast to the impact of land prices on cycles and disparities of real-estate markets. Piazzesi and Schneider (2016), for instance, divide the housing stock in the price of “land” and “physical/built structure” and show that recent price cycles are primarily driven by changes in land prices. Further, in the long-term perspective, Knoll et al. (2017) argue that housing-price increases since World War II have mainly been driven by land and not by the building stock. Beyond cycles, the regional variation of housing prices is also strongly related to land prices. Davies and Heathcote (2007) find that the land share of real-estate prices is about 80% in San Francisco and 12% in Oklahoma City. Differentiating between central and peripheral regions, they identify a positive correlation between the land share of a property price and the level of housing prices. This implies that land prices are a crucial variable for the analysis of housing-price cycles and their spatial patterns. In the following, we discuss the literature on the determinants and disparities of land prices.

### Determinants of land prices

Generally speaking, land prices can be considered the outcome of an interplay between supply, demand, and market institutions. Economic analysis traditionally assumes a perfectly unelastic supply of land (Wiltshaw 1985). Under such conditions, land prices are determined solely by the demand for land, which in turn depends on the amount of money that can be earned by using the land, whereas changes in land prices have no repercussions on the supply of land (Samuelson and Nordhaus 1989). Empirical analysis of zoning policies show that these assumptions are rather unrealistic, because land is a highly regulated commodity and transaction costs as well as planning institutions such as zoning plans regulate the supply of building land (Cheshire and Sheppard 2004). Interestingly, the demand side can have an impact on the supply of building land, as powerful buyers are able to influence the institutions that regulate zoning, in particular when these are institutions on the local scale (Phelps et al. 2021). For decades, local authorities have pursued a generous zoning policy particularly in Austria, converting green and agricultural land to building land: Between 2006 and 2018, Austria’s building-land area increased from 2322 to 3222 km^2^ (Austrian Environmental Agency 2021). As a result, the average annual capital gains due to rezoning in Austria amount to about 2.7 billion Euros (Zens 2011). Building-land reserves as a percentage of total building land are estimated at 25 to 35%, increasing from central to peripheral regions (Musil and Pindur 2008). Consequently, regulation practices in Austria strengthen regional disparities since more restrictive regulation in central regions causes land prices to soar, while the higher elasticity of land supply in the periphery alleviates price pressures.

Location factors influencing land prices can be differentiated in two categories: “first-nature” attributes such as the quality of soil or sun exposure, and “second-nature” attributes, including the connection to infrastructural elements such as road networks or water supplies (Wächter 1993; Belke and Keil 2018). Referring to classic land-use models (e.g. Thünen), Mariš (2021) points out the role of proximity to the CBD, transport connectivity, and in-build infrastructures as relevant factors of changes in land prices. Geographical models of regional land prices focus on the relative position of a region within a spatial system. Drawing upon the work by Thünen and Alonso, the concept of spatial equilibrium provides a general and geographical approach to explain structural variations in regional land prices (Glaeser 2007). The utility of households tends to be equalized across space, because regional disadvantages such as a low level of income or long commuting times are compensated by low land prices, and vice versa. In its basic form, the model predicts a decrease in the price of housing as the distance to employment areas rises (O’Sullivan 2009). Yet, market failures, relocation costs, transaction costs, and the regulation of housing markets introduce frictions which impede market-driven adjustment processes (Cheshire 2013).

Beyond location attributes, land prices are driven by local demand. In contrast to demand-side determinants for agricultural land prices (Ciaian et al. 2012), building-land prices do not figure prominently in the literature. Hence, we discuss the determinants for real-estate prices as proxies. Belke and Keil (2018) identify the number of households and the age structure of the population as important demand-related factors. Geng (2018) finds that a 1% increase in disposable income raises house prices by about 1.5%. Beyond these fundamental demand factors, international and domestic investors engaging in speculative investment (Aalbers 2019) may widen the gap between cities and other areas beyond the variation explained by spatial equilibrium models.

In addition to zoning policies as mentioned above, market institutions in Austria such as a bank-based financial system, a large rental sector in urban areas, legal constraints on securitization measures and an increased awareness of financial authorities regarding financial instabilities after a surge in foreign currency mortgages in the 2000s constitute a relatively stable institutional setup for financing housing demand. Having said this and in line with broader developments in financial markets, a trend toward financialisation, although from a rather low level, can also be identified for Austria (Springler and Wöhl 2020). Interestingly, financialisation has a highly spatially differentiated impact on the demand side: price dynamics in urban agglomerations and international hotspots of tourism and culture are increasingly driven by institutional and international investors, which can cause a decoupling of prices from fundamental demand factors (Van-Hametner and Zeller 2018).

### Disparities of land prices

In regional and urban studies, there is an ongoing debate regarding the issue of price convergences between regional housing markets. Numerous studies analyse various indicators, in particular prices of various building types of housing (e.g. Gray 2018; Hamnett 1988; Blanco et al. 2016), but curiously not of land prices. Some authors stress the impact of housing types (detached, semi-detached, terraced housing, and flats) on the formation of convergence clubs (Holmes et al. 2019), but do not consider the variation of building land.

Empirical studies provide no clear-cut answer on whether regional house prices converge. This can be illustrated for UK house prices (for an overview, see e.g. Gray 2018). For instance, Hamnett (1988) identifies stable house price ranks only in the 1970s, while other authors stress a high stability in price ranks over a longer period (DiPasquale and Wheaton 1996). A long-term analysis for 1973–2009 identifies a pronounced beta-convergence between UK regions in the downward phases of the real estate cycle (Cook 2012). Gray (2018) argues that a key to explaining the inconclusive empirical results might lie in the cyclicality of house prices, as price patterns switch from convergence in a cycle’s downturn to divergence in upturns. Yet, studies for other countries have until now not confirmed a clear correlation between phases of real-estate cycles and a divergence-convergence pattern. Casolaro and Fabrizi (2018) for instance identify five cycles for the Italian regions in the period 1970–2016. While convergence was unrelated to the boom-bust periods for large regions (Northeast, Northwest, Centre, South/Islands), bust periods were associated with convergence within these regions.

Surges in land prices can spill over to other regions and exert an upward pressure on prices throughout the spatial system. According to the literature, there are two different patterns of price diffusion from urban centres (Casolaro and Fabrizi 2018): first, a diffusion that follows the hierarchy of the urban system, and second, the neighbourhood diffusion pattern, caused by short-distance migration and commuters triggering the convergence of housing prices between contiguous regions (Meen 1999; Kuethe and Pede 2011). We subsequently denote the latter pattern as the “ripple-effect hypothesis”. While some authors confirm this hypothesis (for instance, Holly et al. 2011 from London towards the southeast of England, or Holmes and Grimes 2008), others question the local price diffusion trend (e.g. Abbott and De Vita 2013).

## Data and methods

The data source for our study is the “Immobilienpreisspiegel” (WKO 2019), an annual survey published by the Austrian Chamber of Commerce. This database comprises annual building-land prices at the level of districts, measured in Euro per square metre. The period of analysis is 2000–2018 (T = 19) for 95 political districts (*n* = 95), representing the lowest unit of national administration and henceforth are interchangeably referred to as districts or regions. Vienna, which is subdivided into 23 districts, is treated as a single district.^1^ Price data represent average values of registered transactions. They were collected through surveys of realtors. As from 2014, their information is cross-checked with the land registry (WKO 2019). WKO real-estate data have already been used by other studies (e.g. Mundt and Wagner 2017; Van-Hametner and Zeller 2018) and were also integrated in the fundamental price indicator of the Austrian Central Bank (Schneider 2013). Their main advantage is the relatively long time series at the regional level. A comparison of our data with land-price data for Vienna from the Austrian National Bank provides a simple check of validity of our data and reveals that the general trend over the period 2000–2016 is quite similar (+70.1% vs. +73.6%). As a limitation of the WKO-real-estate data it is important to point out that price data refer to land plots of detached houses (sized 600–800 m^2^). Hence, generalizations regarding overall building-land prices must be taken with a grain of salt. As no regional price deflators are available, the empirical analysis uses nominal price data.

In addition to the land-price data, three regional typologies were applied throughout the analysis. These typologies capture crucial supply-and-demand side factors regarding the building-land market: the (1) urban-rural typology, (2) demographic typology, and (3) economic typology. Based on the Austrian urban-rural typology^2^, we identified five settlement types at the district level for our analysis: (i) agglomerations, (ii) suburban regions, (iii) regional centres, (iv) tourism-intensive rural regions, and (v) rural regions. The demographic typology is based on the quartiles of the population-growth distribution of districts between 2002 and 2016. Regions in the fourth quartile are classified as “population-growth high” and the regions in the first quartile as “population-growth low”. Finally, in the economic typology based on Palme (1995), districts are classified by their dominant economic structure as (i) human-capital-intensive (dominance of service sector), (ii) physical-capital-intensive regions (dominance of industry or tourism), or (iii) economic peripheries.

In addition to spatial heterogeneity, which is represented by the three regional classifications, we also account for temporal heterogeneity and distinguish between three sub-periods. These sub-periods are defined by discontinuities in land-price growth rates: 2000 to 2006, a pre-boom period, characterised by stagnant land prices; 2006–2012, an early-boom period with an upsurge in land prices; 2012–2018, a late-boom period which shows even higher growth rates in land prices. The median compound annual growth rate for the three periods increases from 0.68% to 2.85% and 4.33%.

Regional disparities of land prices are measured by sigma and beta convergence. Sigma convergence occurs if disparities among regional land prices decrease over time, whereas beta convergence takes place if land prices in low-price regions grow faster than land prices in high-price regions do (Monfort 2008). The test for sigma convergence is based on the following simple linear time series model (Wieland 2019):

1 $$CV_{i{,}t}=\beta _{0}+\beta _{S}t+\varepsilon _{i{,}t}{,}$$

where *CV*_*i*,*t*_ is the coefficient of variation in region *i* at time *t*. HAC standard errors are used to account for temporal autocorrelation. Beta convergence is tested by regressing the compound annual growth rate of land prices *g*_*P*_ between *t*_0_ and *t*_*n*_ on the initial land price level *P*, i.e.

2 $$g_{P{,}i{,}{t_{n}}{,}{t_{0}}}=\beta _{0}+\beta _{B}P_{i{,}{t_{o}}}+\varepsilon _{i}{,}$$

where index *i* refers to the political district *i*. In order to control for heteroscedasticity due to cross-sectional variability, we calculated a Breusch-Pagan test statistic for each model; in case of a significant result, HC standard errors are applied.

Multiple regression models are applied for the analysis of differences in regional growth dynamics. The empirical cross-section regression OLS models are related to real-estate models of fundamental price determination and conditional convergence models (Belke and Keil 2018). See Sect. 5 for a detailed explanation of the method. Summary statistics for the variables used in the regression models are displayed in Table 1; *gprice* is the explained variable.

**Table 1:** **Descriptive statistics of dependent and independent variables**

Variable	Definition	Source	Minimum	Mean	Maximum	Standard deviation	CV^2^
*gprice*	CAGR (%) of land prices, 2000–2018^1^	WKO	−0.10	2.65	6.34	1.45	0.55
*ghouseholds*	CAGR (%) number pf households, 2000–2018	Statistics Austria	−0.19	0.86	1.88	0.41	0.48
*gincome*	CAGR (%) of annual net income pc of employees, 2001–2018	Statistics Austria	2.58	3.34	3.92	0.30	0.09
*distance*	Travel time in minutes from a weighted average of a regional district’s municipalities to the two nearest federal capital cities	Own analysis	27.67	69.43	138.81	19.05	0.27
*land*	Land reserves as percentage of total construction land (average 2014/2017)	Environment Agency Austria	5.83	24.43	44.84	6.38	0.26
*price2000*	Land price level in 2000 in EUR/m^2^	WKO	21.62	115.60	479.83	91.54	0.79

1: CAGR stands for “compound annual growth rate” which is calculated as $g_{P{,}i{,}{t_{n}}{,}{t_{0}}}=\sqrt[n]{P_{i{,}{t_{n}}}\colon P_{i{,}{t_{o}}}}-1$. 2: CV refers to coefficient of variation

Ripple effects are investigated in Sect. 5 using a cross-autocorrelation analysis as developed by Oikarinen (2004) and deployed, for instance, by Grigoryeva and Ley (2019). The basic idea is to identify potential epicentre regions and estimate the bivariate cross-correlation between temporally lagged price changes in the epicentre region and the regions that are considered receivers of the price signal from the epicentre. Formally, cross-autocorrelation coefficients are a combination of temporal and spatial autocorrelation coefficients and estimated by

3 $$\rho \left({g}_{t-k}^{i}{,}{g}_{t}^{j}\right){,}$$

where ${g}_{t-k}^{i}$ is the land-price growth rate in epicentre region *i* at time *t*−*k* with $k=0{,}\ldots {,}m$ denoting annual lags. The spatial structure is not introduced by a spatial weight matrix but by calculating $\rho$ for regions that satisfy a given set of spatial criteria. For $t=0{,}\, \rho$ measures the correlation of the contemporaneous increase in land prices for regions *i* and *j*, while for $t=1$ the correlation is calculated between land-price growth in the epicentre and land-price growth in the non-epicentre one year later. Arguably, the possibility of a ripple effect is indicted if $\rho \left({g}_{t-k}^{i}{,}{g}_{t}^{j}\right)> 0$ for$k\neq 0$.

## Do land prices converge?

**Fig. 1:**
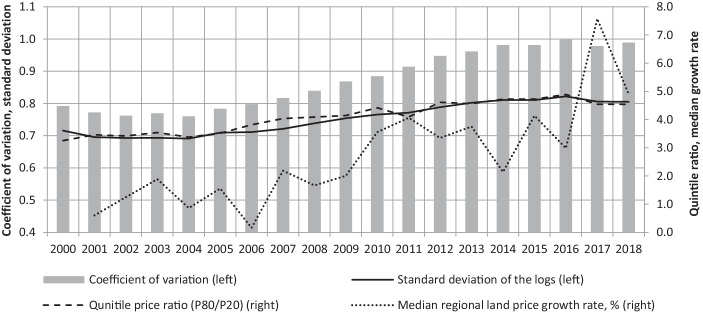
Sigma convergence of land prices in Austria, 2000–2018

Indicators for sigma convergence display a trend towards growing disparities in regional land prices (Fig. 1): the coefficient of variation rose from 0.79 (2000) to 0.99 (2018) – an increase of about 25%. Additional indicators such as the standard deviation and the ratio between the highest- and lowest-price quintile (P80/P20) confirm this general finding. Applying the model in Eq. 1 results in highly significant and positive coefficients ($\hat{\beta }$ = 0.015, *p* value = 0.000), supporting the result of sigma divergence for the period 2000–2018. Beyond this general picture, Fig. 1 reveals a substantial temporal heterogeneity: In the early 2000s, coefficients indicate a slight trend towards convergence, but since 2006, regional land prices have undergone a period of spatial divergence, which lasted until 2014. Since then, the level of spatial price disparities has stagnated for a couple of years and has declined slightly since 2017. In total, the regional land prices seem to follow a cyclical sequence of somewhat declining disparities, sigma divergence, and almost constant disparities.

**Fig. 2:**
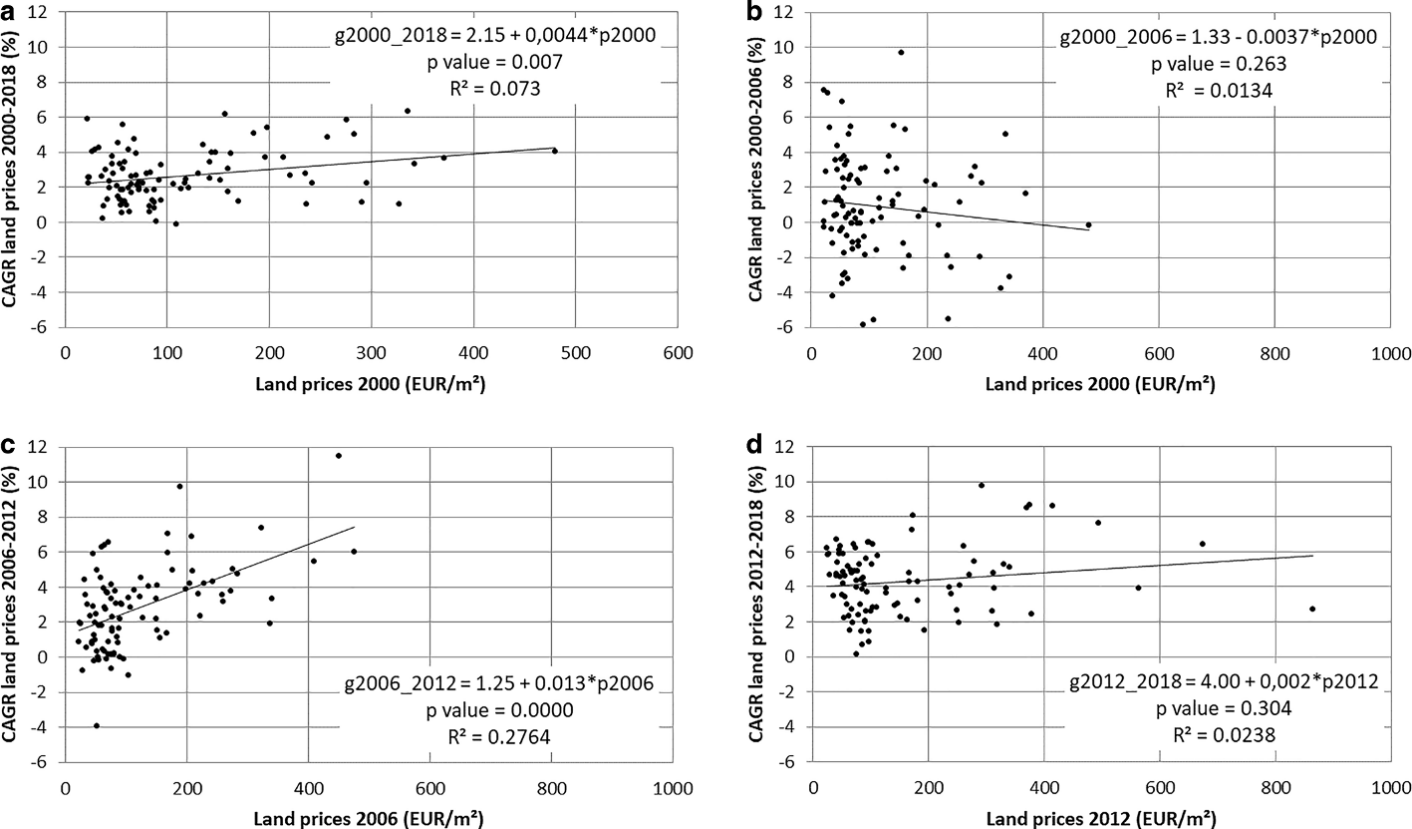
**a**–**d** Beta convergence of land prices in Austria. (Notes: CAGR stands for “compound annual growth rate” which is calculated as $g_{P{,}i{,}{t_{n}}{,}{t_{0}}}=\sqrt[n]{P_{i{,}{t_{n}}}\colon P_{i{,}{t_{o}}}}-1$. “*p* values” refer to the *p* value of the t test of the slope coefficient. Regression results in Panel 4b are based on robust standard errors; Breusch-Pagan tests for models in Panel 3a, 3c and 3d are nonsignificant)

For the three periods under investigation, the analysis of beta convergence broadly confirms the general sequence identified above. In line with sigma divergence, we find statistically significant evidence for beta divergence for the period 2000–2018 (Fig. 2a). A simple linear regression model according to Eq. 2 results in a positive and highly significant slope coefficient. Hence, regions with higher land prices in 2000 experienced a stronger price growth than regions with lower land prices. Like sigma-convergence analysis, the beta convergence also points to a temporal sequence with constant disparities in the first and third sub-periods (Fig. 2b–d) and a statistically significant divergence between 2006–2012, confirming Gray’s (2018) assumption of a cyclical sequence of convergence and divergence.

After having analysed convergence dynamics at the national level, we now focus on dynamics within different types of regions as defined by three regional typologies. The overall spatial development of regional disparities can be understood as a composite effect of convergence/divergence *within* regional subgroups and convergence/divergence *between* regional subgroups. In addition to median land prices and land-price growth rates, Table 2 displays information on sigma and beta convergence. The former is measured by the coefficient of variation, while the latter is evaluated by the slope coefficient from Eq. 2. Regarding sigma convergence, the general trend of rising disparities is corroborated for most types of regions. Except for suburban regions, types of regions with higher land-price growth rates are generally characterized by sigma divergence. Rural and peripheral regions and regions with low population growth rates are characterised by constant regional disparities, i.e., neither sigma convergence nor divergence. These types of regions also stand out because they experience significant beta convergence for the entire period of 2000–2018. Once again, the suburban regions follow a similar but much less pronounced dynamic, which is also expressed by the nonsignificant coefficient of −0.004. While beta convergence is prevalent in the first period under investigation, beta divergence dominates in the period 2006–2012. The beta-divergence trend is particularly strong in economically dynamic regions such as agglomerations, human-capital-intensive regions, or in regions with medium-high population growth.

**Table 2:** **Sigma and Beta-Convergence of regional typologies**

Typology	Category	Median land price 2000 (Euros/m^2^)	Median land price CAGR 2000–2018 (%)^1^	Coefficient of variation (Sigma convergence)					Beta convergence^3^			
				2000	2006	2012	2018	∆2018–2000^2^	2000–2006	2006–2012	2012–2018	2000–2018
Urban-rural typology	Agglomerations	248	3.9	0.475	0.441	0.594	0.571	0.010***	−0.004	0.018**	−0.001	0.005
	Suburban regions	160	3.1	0.544	0.505	0.517	0.560	0.002	−0.015*	0.005	0.000	−0.004
	Regional centre	86	2.2	0.433	0.430	0.481	0.516	0.006***	−0.016	−0.015**	0.002	0.001
	Tourism-intensive rural regions	149	3.1	0.568	0.533	0.626	0.715	0.011***	−0.023*	0.014	0.010**	0.003
	Rural regions	56	2.1	0.362	0.350	0.359	0.338	0.000	−0.044*	−0.007	−0.034**	−0.026*
Demographic typology	Population growth high	173	3.3	0.578	0.549	0.586	0.629	0.005***	−0.010**	0.004	0.002	−0.001
	Population growth medium high	83	2.5	0.667	0.724	0.982	0.979	0.024***	−0.000	0.020***	0.000	0.007*
	Population growth medium low	64	2.0	0.596	0.745	0.793	0.842	0.012***	0.019	0.007	0.004	0.012**
	Population growth low	52	2.1	0.609	0.523	0.575	0.507	0.000	−0.027	0.006	−0.031***	−0.017**
Economic typology	Human-capital-intensive regions	141	3.1	0.636	0.626	0.766	0.772	0.012***	−0.006	0.012***	0.001	0.002
	Physical-capital-intensive regions	109	2.7	0.617	0.622	0.691	0.797	0.012***	−0.007	0.009*	0.010***	0.005
	Economic periphery	54	2.0	0.510	0.468	0.527	0.452	−0.000	−0.036**	0.008	−0.026***	−0.017**

1: CAGR stands for “compound annual growth rate” which is calculated as $g_{P{,}i{,}{t_{n}}{,}{t_{0}}}=\sqrt[n]{P_{i{,}{t_{n}}}\colon P_{i{,}{t_{o}}}}-1$. 2: The numbers represent the coefficient *β*_*S*_ in Eq. 1. 3: Beta convergence is estimated by the coefficient *β*_*B*_ in Eq. 2

Significance levels: *p < 0.1; **p < 0.05; ***p < 0.01

From a spatial planning point of view the pattern is complex and remarkable: Whereas economically dynamic regions become increasingly dissimilar in terms of land prices, relatively underdeveloped or even declining regions are characterised by beta convergence. However, this beta convergence does not lead to sigma convergence, thereby confirming the well-known result that beta convergence is necessary but not sufficient for sigma convergence (Monfort 2008). This finding of rising disparities in high land-price growth regions and constant disparities in low land-price growth regions is reminiscent of the empirical labour economics literature, which argues that unemployment rates converge during economic slumps and rises in boom periods (Werner 2012).

Taken together, land-price dynamics show a sequence of slight convergence, pronounced divergence, and constant disparities, regardless of the “lense” of regional typology that we look through. The finding of constant disparities despite rising land-price growth rates is differentiated by the regional typologies; heterogeneous dynamics might be explained by a process of price-growth diffusion throughout the Austrian land markets (see below). While economic centres and urban regions were the main drivers of price increases and regional divergence, the national price boom did not affect peripheral and rural regions, where converging forces prevailed.

## What determines regional land-price growth rates?

To identify the fundamental factors that explain the growth of land prices in the period 2000 to 2018, a multiple linear regression model is estimated. Concretely, we estimate the following cross-section regression OLS model:

4 $$\textit{gprice}_{i}=\beta _{0}+{x}_{i}^{'}\boldsymbol{\beta }+\varepsilon _{i}$$

where *gprice*_*i*_ refers to the compound annual growth rate of land prices as a percentage in region *i, β*_0_ is the intercept, ${x}_{i}^{'}$ is a vector of the independent variables *ghouseholds, gincome, distance, land, and price2000*, ***β*** is a vector of regression coefficients, and *ε*_*i*_ represents the error term. The variables are defined in Table 1 (see data and methods section). The usual conditions regarding the error term, except the zero conditional mean assumption, are assumed (Verbeek 2008). As a result, the ceteris paribus interpretation refers only to the variables that are included in model (4) and a causal interpretation is not warranted (Arkes 2019). Due to the assignment of competencies for land-use regulation to the level of the nine federal states, observations for political districts are not independent and the error term *ε*_*i*_ may not be $i.i.d.$ As a remedy, model (4) is estimated with standard errors clustered at the level of the federal states. Furthermore, estimation of unweighted and weighted regression models (regional population in 2018 is used as weight) accounts for the substantial variation in terms of size between the regions.

The covariates capture the demand (*ghouseholds, gincome*) and supply side (*land*) factors of the land market. Growing numbers of households and rising incomes are expected to have a positive effect, while increased land supply should dampen land-price dynamics. Because of data limitations, the variable *land* does not enter our model as a growth rate; it is assumed that the distribution of *land* approximates structural differences in land reserves between urban and rural regions. The variable *distance* is intended to measure the centrality of a region and *price2000* controls for initial land-price differences. Increasing distance to economic centres is assumed to affect land-price growth negatively. Bivariate correlation coefficients for the independent variables are all below 0.6, suggesting that problems with multicollinearity may be limited. Interestingly, income growth shows a negative correlation with *gprice* (−0.27).

Regression results are presented in Table 3. Columns (4.1) and (4.2) are estimations of model (4) with and without weighting, while columns (4.1out) and (4.2out) present re-estimations of the models (4.1) and (4.2) without outliers as defined by Cook’s distance which happen to be three in both instances.^3^ The F‑statistic is statistically significant for all specifications and the R^2^ ranges from about 16% for model (4.1) to 30% for model (4.2out), suggesting that factors specific for individual regions play an important role in explaining land price growth rates. Weighting the observations by population improves the models’ fit by about 76%. Test statistics for heteroskedasticity, spatial autocorrelation, and functional misspecification and normality of the error terms suggest that the model assumptions hold, and that the results are valid; the only diagnostic test which is significant at the 10% level is the RESET test for model (4.1out).

**Table 3:** **Regression results (OLS)**

	*Dependent variable*			
	CAGR rate of land prices (%), 2000–2018 (gprice)			
	(4.1)	(4.2)	(4.1out)	(4.2out)
*ghouseholds*	0.718**	0.956***	1.213***	1.230***
	(0.358)	(0.317)	(0.419)	(0.461)
*gincome*	−1.156*	−0.863*	−1.132**	−0.813**
	(0.604)	(0.515)	(0.552)	(0.387)
*distance*	0.015	0.024*	0.027***	0.025**
	(0.011)	(0.012)	(0.009)	(0.014)
*land*	−0.065*	−0.099**	−0.059**	−0.109**
	(0.033)	(0.040)	(0.028)	(0.047)
*price2000*	−0.001	−0.002	−0.001	−0.002
	(0.003)	(0.002)	(0.003)	(0.003)
Constant	6.549***	5.729***	6.818***	6.187***
	(2.176)	(1.876)	(0.859)	(0.818)
Weighting (Population 2018)	No	Yes	No	Yes
Observations	95	95	92	92
R^2^	0.162	0.285	0.206	0.296
Adjusted R^2^	0.115	0.245	0.160	0.255
F Statistic	3.447***	7.099***	4.463***	7.233***
Breusch Pagan test (*p* value)	0.427	0.427	0.3915	0.4188
Moran’s I test for spatial autocorrelation (*p* value)^1^	0.182	0.109	0.283	0.143
RESET test (*p* value)	0.524	0.524	0.077*	0.476
Kolmogorov-Smirnov test (*p* value)	0.324	0.466	0.662	0.762

Clustered standard errors at the level of federal states in parentheses; 1: Spatial weights matrix: Contiguity based on queen criterion. Equation (4.1out) and (4.2out) repeat Euqation (4.1) and (4.2) after 3 outliers have been removed from each regression model

Significance levels: *p < 0.1; **p < 0.05; ***p < 0.01

In the following, we discuss the explanatory value of independent variables for model (4.1). On the demand side, growth of households has a positive and significant association with land prices. The partial effect of a one-percentage-point increase of *ghouseholds *for given levels of *gincome, distance, land*, and *price2000* represents an average 0.718 percentage point increase in the growth rate of land prices, which is about 0.5 standard deviations of *gprice*. Having said this, the estimated coefficients for *ghouseholds* are likely to underestimate the causal effect of the growth of households on land prices because a surge in land prices due to an exogenous rise in households will likely lead to the displacement of poorer households, thereby dampening the upward pressure on land prices. Income growth shows a negative association with land price growth. This finding is remarkable, as it points to looming social problems regarding the affordability of housing. Households in regions with lower income-growth faced higher land-price growth rates, a finding which is true for agglomerations such as Vienna, Salzburg, or Innsbruck, i.e., regions that are in the focus of public debates because of their expensive housing markets (Zoidl 2021). This finding of a negative correlation between income growth and land-price growth may be biased upwards or downwards in two ways: Firstly, a selection effect could be at work whereby low-income households move from rising land-price areas to less expensive regions, resulting in a downward bias. Secondly, an influx of refugees into urban areas, an omitted variable in our model, might result in higher prices and lower income and hence in an upward bias of our estimated coefficient for *gincome*. Below we explore the relationship between income and land prices in more detail.

On the supply side, building-land reserves show the expected negative sign and a significant coefficient. This finding is corroborated by the fact that the share of building-land reserves increases towards rural and peripheral regions (Musil and Pindur 2008; Banko and Weiss 2016). In contrast, *distance* does not show the expected negative sign and only the weighted model and the models excluding the outliers display significant coefficients. Hence, land-price dynamics do not follow a simple central-periphery logic, as regions beyond agglomerations and their suburbs likewise experienced substantial price surges. Presumably, tourism-intensive regions are the main drivers of these spatially heterogeneous growth patterns of land prices. The land price in 2000 shows a nonsignificant coefficient, validating the identified absence of beta convergence for Austrian land prices. Standardized coefficients reveal that *land* (−0.28) and *price2000* (−0.06) have the largest and the smallest effect size in model (4.1).

The regression results were subject to several robustness checks. First, we tested for the relevance of additional regressors, namely the availability of infrastructure as measured by hospital beds per 100,000 inhabitants and number of students at universities of applied sciences per 1000 inhabitants. Yet, these variables are nonsignificant and their inclusion does not change the main results. The same holds true for the inclusion of regressors to capture nonlinear effects of distance on price. Second, we used different spatial weights (distance-based measures as opposed to a spatial neighbourhood criterion) to test for spatial autocorrelation of the error terms. Excepting one test, all results were nonsignificant. Third, quantile regressions for the first and third quartile disprove the existence of non-uniform effects across the distribution of the dependent variables. Fourth, we re-estimated the models (4.1) and (4.2), excluding outliers according to Cook’s distance measure. This resulted in the exclusion of three albeit different observations for both models. Compared to the models with all observations, there are no changes of signs, while the level of significance tends to increase. The size of coefficients changes only marginally after the removal of outliers in model (4.1out) and (4.2out). Taken together, the robustness checks strengthen the presumption regarding the validity of our findings.

The regression analysis suggests that an increase in income growth is associated with a decline in land price growth rates. Considering the social relevance of the affordability of land, this finding merits further exploration. At one end of what we may call the affordability distribution are regions with average annual land price growth rates outpacing average annual income price growth rates, which results in deteriorating affordability of land by the local population. Two extreme examples are Salzburg Stadt and Kitzbühel, the former being a tourism-intensive agglomeration, the latter being a tourism hot-spot with strong external demand for land. The average annual change in land prices for the period 2001–2018 was 6.7% for both regions, while the increase in income lagged behind with a growth rate of 2.9% (Salzburg Stadt) and 3.5% (Kitzbühel). For comparison, median changes in land price amounted to 2.6% and in income to 3.4%. Vienna, which is often at the centre of the public debate on affordability of housing, experienced a 4% land price growth rate and a 2.7% income growth. The fact that Vienna’s income growth is below the national average indicates some structural and institutional problems of a modern urban economy with many low-paid service sector jobs (Riesdenfelder et al. 2011; see Henning and Eriksson 2021 for a general overview on labour market polarisation). At the other end of the affordability distribution are regions with land price growth rates falling short of income growth rates, which results in an improvement in the affordability of land. Freistadt and Lienz, two regional centres, are among the regions with low land price growth rates (0.3% and 0.5%) but relatively high income growth rates (3.9% and 3.8%). A strong manufacturing sector within the region or nearby combined with powerful unions might explain the superior income development in Freistadt and Lienz, while the dominance of the tourism sector may account for the low pay rises in Salzburg and Kitzbühel. Hence, the affordability of land is a complex outcome of the interaction of internal and external demand for land, land supply and income dynamics, which are in turn related to structural characteristics of the regional business sector.

## Spatial diffusion of the land-price boom

According to the ripple-effect hypothesis, land-price changes in a region are affected by price changes in “epicentre” regions, which spill over to neighbouring regions (Meen 1999, 2016; Holly et al. 2011). An alternative diffusion of land-price impulses follows the urban hierarchy from first- to second- and third-tier cities, independent of spatial proximity. To discriminate between the two explanations, we compare the annual land-price growth rates between different settlement types and analyse cross-correlations of temporally lagged land-price growth rates between epicentre regions and other regions.

An analysis of average annual land-price growth rates for Vienna and the regions according to the urban-rural typology confirms the role of agglomerations as price drivers: in the first period (2000–2006), Vienna and the other agglomerations experienced the highest growth rates, besides touristic regions. In this first period, polycentric impulses seem to be at work, also originating in agglomerations beyond Vienna. The growth dynamic of agglomerations accelerated further in the second period (2006–2012), but suburban regions, regional centres, and peripheral regions likewise experienced increasing growth rates. In the last period (2012–2018), growth rates indicate a spatial shift suggesting the presence of a ripple effect: whereas the growth rates of agglomerations declined, they remained high or increased even further in suburban regions and in the other regional types. This pattern can be interpreted as a combination of both mechanisms of diffusion. Patterns of touristic regions however follow a different logic, possibly because land prices in those regions are mainly determined by extra-regional demand.

As a second approach, we apply the cross-autocorrelation analysis of Oikarinen (2004), with quadruple lags. Table 4 shows the results for the price diffusion along the urban hierarchy. The findings above and the literature suggest the choice of Vienna (VIE) and the agglomerations excluding Vienna (AGG) as our two epicentres of interest. Almost all coefficients are positive, illustrating common trends and spatial spillovers of land-price dynamics. Evidence for a price diffusion along the urban hierarchy is provided by strong correlations between Vienna, the agglomerations, and the suburban regions for *t* = 0,1,2, whereas rural regions display the highest impact after four-time lags. Price developments in touristic regions defy a simple interpretation, confirming once again the idea that their dynamics are related to other factors such as international demand. In Table 4, the right side displays the role analyses of agglomerations, excepting Vienna, as potential epicentres. Positive and significant coefficients for suburban regions excluding the Viennese suburbs (t-2), regional centres (t-2), the touristic periphery (t-2), and rural regions (t-4), corroborate the finding of a polycentric pattern of epicentres for Austrian building-land markets.

**Table 4:** Cross-correlations between lagged annual price changes at epicentres and annual price changes in different types of regions

	VIE t-0	VIE t-1	VIE t-2	VIE t-3	VIE t-4	AGG t-0	AGG t-1	AGG t-2	AGG t-3	AGG t-4
Vienna (VIE)	–	–	–	–	–	0.676*	0.433*	0.554*	–0.094	–0.158
Agglomeration (excluding Vienna) (AGG)	0.676*	0.547*	0.347	0.512*	0.334	–	–	–	–	–
Suburbia of Vienna	0.396	0.283	0.623*	0.280	0.238	0.564*	0.474*	0.204	0.138	0.394
Suburban region (excluding suburbia of Vienna)	0.683*	0.395	0.480*	0.494*	0.016	0.712*	0.415*	0.805*	0.262	0.205
Regional centre	0.309	0.220	0.326	0.324	0.395	0.607*	0.220	0.481*	0.194	0.421
Touristic periphery	0.427*	–0.001	0.272	0.108	0.211	0.184	0.006	0.485*	0.116	0.450
Rural region	0.281	0.040	0.161	0.278	0.499*	0.330	0.220	0.241	0.222	0.567*

*Numbers with an asterisk display significant correlations at the 10% level of significance

**Fig. 3:**
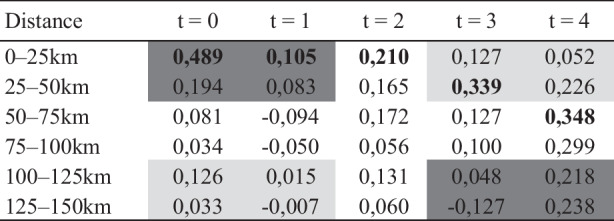
Average Cross-correlations between lagged annual price changes in Vienna, Graz, Linz and Salzburg and annual price changes in districts within the distance of 0–125 km. The cells display average cross-correlation coefficients. Bold numbers refer to the respective column maximum

Cross-autocorrelations between the four largest Austrian agglomerations (Vienna, Graz, Linz, Salzburg) and other regions located within certain distance bands from Vienna are applied to study the ripple-effect hypothesis. Fig. 3 is constructed by using a space and time variable and summarizes the results for the four agglomerations. About 83% of the cells contain positive values, which is in line with theoretical expectations: price changes in one region induce price changes in the same direction in adjacent regions. The rather low values of the cross-autocorrelation coefficients are primarily the result of the small values for the cities of Salzburg and Graz, while Vienna and Linz display considerably higher coefficients. For the city of Salzburg, this likely has to do with the specific spatial structure of districts and the presence of tourism-intensive districts, the price dynamics of which are influenced by external forces of demand and not by price impulses emanating from the city of Salzburg. Regarding Graz, subdued land price increases than in the other three cities and low population growth in Styria are likely to reduce the transmission intensity of price signals.

In addition, the maximum value within each column of Fig. 3 tends to be at a higher distance value as the time lag increases. This pattern also corroborates the ripple-effect hypothesis as the price impulses from the epicentres travel through time and space. Evidence from t = 4 at Fig. 3 might suggest that the ripple effect ends between 50 and 100 km. Once again, however, this hides important differences between the four cities. Price impulses from Vienna and Linz travel as far as 150 km, while ripple effects for Graz and Salzburg are much more limited in space. Tabular analysis can also be used to conduct a simple numerical test of the ripple-effect hypothesis. This test, which is of course not a significance test, is based on the following insight: If this hypothesis holds true, the average of the values in the upper left and lower right cells of the table (dark grey cells in Fig. 3) should be larger than the average of the values in the lower left and upper right cells (light grey cells in Fig. 3). That is, cross-correlation coefficients for low values of t (t = 0,1) and a close distance to the agglomerations (0–50 km) should be positive and relatively larger as compared to coefficients for high values of t (t = 3,4) and further away from agglomerations (100–150 km). Applying this method to the four cities^4^ as well as to Fig. 3 reveals that the average of the dark grey cells outweighs the average of the light grey cells, thereby suggesting the presence of a ripple-effect.

Taken together, the empirical analysis indicates some evidence of the ripple-effect hypotheses, albeit with a substantial variation among the four agglomerations.

## Discussion and conclusions

This paper investigates the regional variation of the Austrian land-price boom between 2000 and 2018. Sigma and beta convergence analyses show a divergence trend for the whole observation period, suggesting that the price boom occurred parallel to increasing disparities. For the three sub-periods 2000–2006, 2006–2012, and 2012–2018, one may observe a cyclical sequence of slight convergence, significant divergence, and constant disparities. The analysis of building-land prices according to regional typologies shows that regions with low price-growth rates, such as the economic periphery or rural regions, have converged, while regions with higher price-growth rates such as agglomerations, tourism-intensive regions, or human-capital-intensive regions experienced diverging regional land prices. Furthermore, between the first and the third period, regional analysis reveals an increasing heterogeneity of patterns of convergence and divergence between different regional types. Overall, the analysis provides some support for Gray’s (2018) hypothesis of divergence during high price-growth episodes and convergence in periods with low land-price growth rates.

Regional land-price growth is positively correlated with growth in the number of households as well as with distance from the federal capital cities. Positive coefficients for distance, which however are not significant in all models, suggest that land-price growth patterns in Austria do not follow a simple gradient from the centre to the periphery. Presumably, the presence of high growth rates in the touristic regions outside urban centres accounts for this finding. While the negative effect of land reserves is in line with expectations, the negative result for the income parameter is surprising. A causal interpretation of the income effect would be misleading, but the negative association might indicate a decoupling of land prices from household income, compounding the problem of declining affordability of housing in urban and touristic regions. Considering the interplay between nationally determined income growth rates and regional land-price dynamics could add important insights for housing as well as social policy makers.

Regarding the spatial diffusion of rising land prices, results point to a ripple effect combined with a diffusion alongside the urban hierarchy. As such, the land-price boom originated simultaneously in the polycentric urban system and then spread to neighbouring suburban regions and finally to other regions. Touristic regions follow a different path as their land-price dynamics are heavily affected by extra-regional demand-side factors. We conclude that land-price dynamics in Austria do not follow a simple centre-periphery diffusion, as other studies would suggest (Holly et al. 2011).

Taken together, this research contributes to international debates on land prices. It does so by adding a nuanced perspective from an economy with a specific institutional setup, economic structure, and historical development. The paper demonstrates how the hidden complexities and intricacies of land-price dynamics in Austria are revealed by a context-sensitive and detailed spatial analysis. One-size-fits-all explanations and a narrow focus on selected urban agglomerations are called into question.

Regarding limitations, it is important to point out that our land price data represent prices for land plots of detached houses (sized 600–800 m^2^). This implies that they are more representative for land price dynamics in rural and suburban regions, where detached houses play a more relevant role in the housing market. In terms of affordability, land prices for public housing and multi-storey buildings are crucial but not included in our data set. Taken together, these limitations should caution against sweeping generalizations and strong policy conclusions based on the presented findings.

This paper has several implications for future research: first, it demonstrates that the cyclicality of land prices has an important explanatory value for regional disparities in land prices. Secondly, land-price booms do not only affect urban agglomerations. Hence, research as well as policies should devote more attention to regions beyond the main agglomerations. The Austrian case suggests that a decoupling of land prices from household income can also take place in non-urban areas, a finding which might retain its relevance, not least because of changes in land demand due to the Covid-19 pandemic.

## Footnotes

1 Price data for Vienna cover only the larger, peripheral districts. Inner-city districts’ land prices are not given, as almost no building land is available.

2 The Austrian urban-rural typology identifies 11 settlement types, based on settlement structure, traffic accessibility, and the role of tourism: https://www.statistik.at/web_de/klassifikationen/regionale_gliederungen/stadt_land/index.html (09.09.2019).

3 The outliers according to Cook’s distance for model (4.1) are Salzburg (Stadt), Lienz and Lilienfeld; for model (4.2) the outliers are Graz (Stadt), Salzburg (Stadt) and Gänserndorf. Cook and Weisberg (1999) suggest that observations with a Cook’s distance > 0.5 represent possible outliers, while cases with Cook’s distance > 1 should always be inspected in greater detail since they represent severe outliers. Yet, there are alternative rules which suggest lower threshold values, such as 4/n, should be used. In this study we applied a pragmatic and intermediate approach between these threshold values. All Cook’s distances in this analysis are below 0.2. The three observations with the largest Cook’s distances in model (4.1) and (4.2) have been identified as outliers. Their distances are between about 0.1 and 0.16. The plot function for regression diagnostics provided by R was used to identify outliers.

4 The individual tables for the four cities are not displayed.
